# Institutional profile: the national Swedish academic drug discovery & development platform at SciLifeLab

**DOI:** 10.4155/fsoa-2017-0013

**Published:** 2017-04-07

**Authors:** Per I Arvidsson, Kristian Sandberg, Kjell S Sakariassen

**Affiliations:** 1 Science for Life Laboratory, Drug Discovery & Development Platform & Division of Translational Medicine & Chemical Biology, Department of Medical Biochemistry & Biophysics, Karolinska Institutet, Stockholm, Sweden; 2 Catalysis & Peptide Research Unit, University of KwaZulu-Natal, Durban, South Africa; 3 Science for Life Laboratory, Drug Discovery & Development Platform & Organic Pharmaceutical Chemistry, Department of Medicinal Chemistry, Uppsala Biomedical Center, Uppsala University, Sweden; 4 Physiology & Pharmacology, Karolinska Institutet, Stockholm, Sweden; 5 KellSa s.a.s., Str. Campo e Zampe 12, I-13900 Biella, BI, Italy

**Keywords:** academic drug discovery, academic medical research, antibody therapeutics, biopharma, drug development, drug discovery, pharma research, small molecules

## Abstract

The Science for Life Laboratory Drug Discovery and Development Platform (SciLifeLab DDD) was established in Stockholm and Uppsala, Sweden, in 2014. It is one of ten platforms of the Swedish national SciLifeLab which support projects run by Swedish academic researchers with large-scale technologies for molecular biosciences with a focus on health and environment. SciLifeLab was created by the coordinated effort of four universities in Stockholm and Uppsala: Stockholm University, Karolinska Institutet, KTH Royal Institute of Technology and Uppsala University, and has recently expanded to other Swedish university locations. The primary goal of the SciLifeLab DDD is to support selected academic discovery and development research projects with tools and resources to discover novel lead therapeutics, either molecules or human antibodies. Intellectual property developed with the help of SciLifeLab DDD is wholly owned by the academic research group. The bulk of SciLifeLab DDD's research and service activities are funded from the Swedish state, with only consumables paid by the academic research group through individual grants.

## Science for Life Laboratory

Science for Life Laboratory (SciLifeLab) [1] is a national Swedish research infrastructure with two nodes, one in Uppsala and one in Stockholm. Started in 2010 by the four universities in the Stockholm/Uppsala area: Karolinska Institutet, KTH Royal Institute of Technology, Stockholm University and Uppsala University, the organization became a national research infrastructure in molecular bioscience in 2013 [2,3]. Since 2016, the infrastructure has expanded its footprint to all other major Swedish universities and now combines frontline technical expertise with advanced knowledge of translational medicine and molecular bioscience with a focus on health and environment. Researchers from all of Sweden can access the technology and the know-how available at SciLifeLab for their research projects; users are found within academia, industry, authorities and healthcare. In addition, SciLifeLab aims to create a strong research community through workshops, seminars and courses. SciLifeLab is divided into a number of platforms that cover a large part of the biomedical value chain from ‘bench-to-bedside’ starting with genomic sequencing [4], chemical biology [5], drug discovery [6], and ending with patient stratification through clinical genomics; in addition, advanced imaging and proteomics techniques [7] are made available. Thus, SciLifeLab has a unique position to promote partnerships and mediate collaborations between actors in the life science sector.

## SciLifeLab DDD

The SciLifeLab Drug Discovery and Development (DDD) consists of ten expert facilities which provide an industry standard infrastructure and expertise, providing strategic support for academic study projects to reach a preclinical proof-of-concept [6]. The facility scientists have extensive experience from both academy and biopharma/pharma organizations. The platform directors are Per I. Arvidsson, Karolinska Institutet, Stockholm, and Kristian Sandberg, Uppsala University, Uppsala, Sweden.

The current services offered by the SciLifeLab DDD are:

- Up to 80 h free support to prepare and transform a project idea into a drug discovery/development project. The support includes preparation of a first draft of a drug target product profile and a lead generation/antibody selection strategy with progression plans;
- Support in initial toxicity assessments and in the design of pharmacological *in vivo* studies;
- A strategic collaboration with SweTox [8], concerning drug safety solutions such as, *in silico*, *in vitro* and *in vivo* capabilities;
- In collaboration with the Chemical Biology Consortium Sweden [5], easy access to a large compound collection;
- Protein and cell line production for assay development;
- Identification of binders from the SciLifeLab DDD intellectual property (IP) free phage display library of human antibodies;
- Medicinal-, synthetic- and computational chemistry;
- Biochemical and cellular assays, both at single concentration to determine activity in large compound libraries and to generate dose response data for iterative chemistry;
- Biophysical characterization of molecular interaction between target and compounds or antibodies;
- Support in initial toxicity assessments and in the design of pharmacological *in vivo* studies;
- Analysis of metabolism of and pharmacokinetics (PK) for compounds and antibodies;
- Advanced analysis of *in vitro* pharmacology and systems pharmacology of compounds and antibodies.

Importantly, in agreement with the Swedish teacher's exemption law, the scientist retains all rights and ownership during this process. The functions, expertise and key publications of the ten expert facilities are described in more details below.

## Target product profiling & drug safety assessment

This facility gives support to principal investigators and innovation agencies in preparing a first target product profile, assists with platform and project coordination, and gives assessment of target dependent toxicity and support with the design of pharmacological studies *in vivo*. Two full-time platform directors lead the work in Stockholm and Uppsala, respectively. One project coordinator with extensive experience in pharmacological studies supports the projects. This virtual facility also has access to one drug discovery toxicologist via a strategic collaboration with the Swedish Toxicology Sciences Research Center (Swetox) providing expert consultation using literature/databases for safety assessment of targets and compounds. Analytical support on the pharma industry is provided via Informa CiteLine^® and^ Trial Trove^®^ (London, UK) and Thomson Reuters Integrity^®^ and Cortellis^®^ (Toronto, Canada).

## Compound center

The compound center facility distributes compounds to principal investigators in assay ready plates from the SciLifeLab compound collection. Two scientists are working with maintenance and the distribution of compounds from the SciLifeLab compound collection, approximately 200,000 chemical substances. The team is also working to establish a national IT infrastructure (the commercial CDD – collaborative drug discovery is currently being used) to store information about the compounds effect on biological properties that would allow easy access for the project owner.

## Protein expression & characterization

Service is given to academic groups engaged in drug discovery projects, in other words, supply of proteins and cells as well as scientific and technical support, for example, access to skills, lab facilities and instrumentation. The end products are well characterized proteins and cells fit for purpose for use in drug discovery projects within the DDD Platform. This includes material for assay development, screening, mode of action studies and structure determination as exemplified in these references [9,10]. In addition, development and establishment of technologies and methods relevant for protein expression, purification and characterization is performed within this facility. One academic professor acts as Facility Director and three full time scientists with industrial experiences are running the lab. The facility has a fully equipped lab for protein expression and purification. The instrumentation includes, for example, qPCR instrumentation, isothermal calorimeter, biosensor instrumentation, and a liquid handling robot.

## Biochemical & cellular screening

Set up of validated primary assays and orthogonal assays with sufficient throughput to support drug discovery programs with single concentration data for screens up to 30,000 compounds and dose-response data for selected and/or synthesized compounds to guide chemistry is provided from the biochemical and cellular screening facility [11,12]. The team also supports antibody programs with orthogonal assay data for binders from phage display libraries. One academic professor acts as Facility Director and the facility is run by four full time scientists with industrial experiences. The facility has a fully equipped cell room for cell cultivation. The instrumentation includes instrumentation for ultrasonic non-contact dispensing, a medium throughout robotic liquid handler, plate readers, cell washers and multichannel dispensers.

## Human antibody therapeutics

The human antibody therapeutics facility performs selection and characterization of therapeutic antibody candidates from IP-free human phage libraries [13], and produces scFvs and IgGs for *in vitro* experiments. The selection is followed by initial characterization of the isolated antibodies: DNA/amino acid sequence, binding specificity/selectivity, affinity, stability, aggregation, epitope mapping, among others. [14]. Additional services include design and production of novel binders, for example, bispecific antibodies and humanization of murine antibodies; support in outsourcing production of large quantities of antibodies; assessment of function and biological potency of the antibodies using *in vitro* assays; consulting at the preproposal stage with potential partners on project design among others; collaboration in the design of relevant assays for biological potency of the antibodies and in designing preclinical proof-of-concept studies. Three academic professors act as Facility Directors. The facility is staffed with four scientists working at SciLifeLab Stockholm and two scientists at the U-Read lab at Lund University (U-read) [15]. Both labs are fully equipped for molecular biology work and phage display technology, including instrumentation allowing semi-HTP selection and screening of antibody expressing clones in a variety of immunoassay platforms including ELISA, HTRF, surface plasmon resonance and a high-throughput flow cytometer assay platform for analysis of protein binding to cells and particles. Also, since the scientists in Stockholm are co-localized with the Protein Expression and Characterization facility (see above), the facility has access to the same instrumentation park.

## Biophysical screening & characterization

Identification and characterization of ligands, structural biology and fragment-based lead generation is provided by the biophysical screening and characterization facility [16–18]. Key technologies are: surface plasmon resonance (SPR) for ranking, kinetic analysis (Kd, dissociation rate), target engagement, fragment screening; microscale thermophoresis for affinity (Kd) ranking, target engagement and; x-ray crystallography for studies of detailed ligand–protein interactions, structure-based hit or lead optimization and target engagement [19]. The biophysical screening and characterization facility also gives consultations on the above techniques, set up for validated assays and help to analyze ligand interaction with protein structures. Two academic professors act as Facility Directors and the facility is staffed with three scientists with extensive expertise in biophysics, biochemistry, structural biology and drug discovery. The instrumentation includes: SPR instrumentation, microscale thermophoresis equipment, automated liquid handler, a crystallization robot, a plate hotel, dynamic light scattering (DLS) instrument and various protein analysis instruments.

## ADME of therapeutics

The focus for the ADME of therapeutics (ADMEoT) facility is to determine absorption, distribution, metabolism and excretion (ADME), and perform pharmaceutical profiling of small molecule compounds and antibodies [20–23]. The ADMEoT facility services include giving access to expertise and know-how to the project teams with regard to identifying critical points for drug discovery, study planning, performing wet lab studies and support to principal investigator in ADME/PK questions in the project and in discussions with potential investors and regulatory bodies. The facility performs in silico and *in vitro* measurements to characterize PKPD properties of compounds and antibodies, performs bioanalysis from *in vivo* studies, basic formulation of compounds and pharmacokinetic and pharmacodynamic (PKPD) modeling. One academic professor acts as Facility Director and the facility is run by four full time scientists with industrial experiences and expertise knowledge in physicochemistry, analytical chemistry, drug metabolism, physiologically-based pharmacokinetic, PK, PK/PD modeling, drug discovery process for small molecules and biologics, project leading and management. Instrumentation includes one mass spectrometer and a liquid handling robotic system. Core instruments for all activities at the facility are the mass spectrometer, liquid chromatography (UPLC-MS/MS) and liquid handling robotic system. The mass spectrometer is an analytical instrument with a high degree of flexibility used to address most of the chemical space of compounds encountered by the facility: small molecules, peptides and biologics. The facility also has access to additional equipment at the academic setting. This includes an additional UPLC-MS/MS mass spectrometer, liquid handling robotic system, a biosafety level 2 cell culture laboratory with attached equipment and a pKa determination apparatus.

## *In vitro* & systems pharmacology

The *in vitro* and systems pharmacology facility performs investigation of drug mechanism of action for product differentiation and identification of putative biomarkers using a systems pharmacology approach to *in vitro* efficacy studies including assay development and drug combination studies. Special techniques include primary cells from patients, preparation culturing and testing in various cell assays [24], 3D-cell culture techniques in 384-format [25], transcriptomics screening in 384-format (in collaboration with Justin Lamb) [26], Cmap analysis [27,28], imaging, time-lapse, studying confluence, apoptosis, growth patterns, morphology, subcellular structures [29,30], drug combination studies on viability/proliferation, differentiation, subcellular morphology. Multivariate data analysis includes machine learning and mass spectrometry-based metabolic and proteomic profiling. Two academic professors act as Facility Directors. The facility is run by four scientists with experience from academia, pharma industry, start-ups, clinical chemistry and pharmacology university hospital and expertise knowledge in various aspects of computational medicine and medical bioinformatics. The following instruments are shared with the Uppsala University hospital clinical pharmacology laboratory: Acoustic dispenser; High-throughput screening platform, with a robotic arm, washer and dispenser; several multimode plate readers (luminescence, fluorescence, absorbance, label free); time-lapse imaging systems; automated fluorescence microscopy, automated western blot system, ab-based multiplex protein quantification system, and equipment for image cytometry. Through collaboration with Uppsala University Hospital Clinical chemistry lab, the facility also has access to a high-resolution mass spectrometer with nano-LC system.

## Medicinal chemistry – Hit2Lead

Services from the Hit2Lead facility is the quality control of the initial active compounds and further design and synthesis of compounds suitable for drug discovery [31,32]. Quality control of the initial data during hit-lead generation includes verification of the hit compounds identified during the lead generation activities. This includes verification of the structure, the purity of the compound, that it is not interfering with the assay (PAINS) and that there is a true interaction with the biological target, etc. Design of compounds suitable for drug discovery includes addressing all unforeseen problems a molecule may encounter on its way toward the market. This means finding the right balance of potency, metabolic stability, permeability, safety, among others. Synthesis of the compounds is of course a prerequisite to progress the project. Often the compound design challenges the synthetic chemists to explore new areas of organic chemistry. This facility works closely and coordinates all their activities with the facility Medicinal Chemistry – Lead Identification at Uppsala University. One academic professor acts as Facility Director and five full time scientists with industrial experiences and expertise in synthetic chemistry and computational chemistry are engaged in the project work. The lab is equipped for organic synthesis including chemicals, microwave reactor, hydrogenation apparatus, NMR 400 MHz, LC/MS, prep LC, and semi-prep supercritical fluid chromatography for chiral chromatography. Computer hardware and licenses for software programs necessary for ligand and structure-based design, as well as software for virtual screens. Through the compound center (see above), the facility also has access to a unique compound collection of about 200,000 compounds.

## Medicinal chemistry – lead identification

The medicinal chemistry – lead identification facility, localized to Uppsala University, optimizes the lead series identified by the MCH2L facility at Stockholm University to deliver molecules with improved properties to meet the demands for a desired biological effect, described by the candidate drug target profile [33–35]. The process includes iterative design, synthesis and analysis of assay data to improve compound properties. One academic professor acts as Facility Director and three full time scientists operate the facility. All chemists have a PhD degree with a broad industrial and academic experience in designing drug molecules and develop and apply synthetic methods to deliver molecules for biological profiling. Competence for PET-radiochemistry is also available within the facility. Well-equipped labs for organic synthesis are available at the Division of Organic Pharmaceutical Chemistry, Uppsala University, including NMR-spectrometers, preparative LC, automated flash instruments, UPLC-MS, peptide synthesizers, microwave reactors, and flow reactors.

## Delivering de-risked research programs for partnering

**Figure 1.**
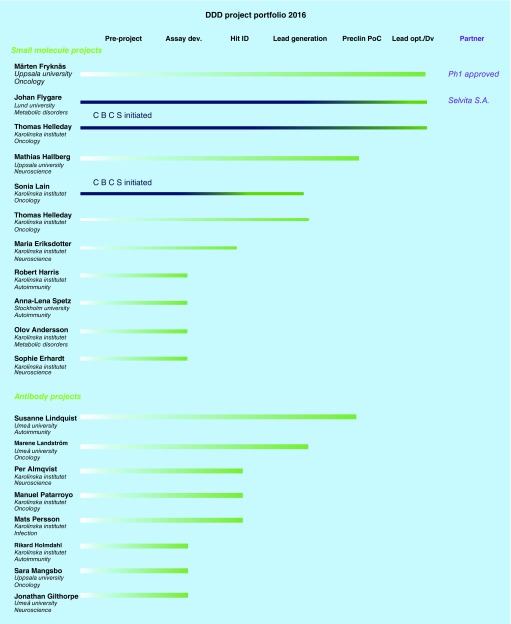
**A list of the academic researchers and projects that have received support from the SciLifeLab platform during 2016 after evaluation by external platform steering group.** Marked in blue are projects that were initiated at the Chemical Biology Consortium Sweden – a separate but closely collaborating ‘sister’ platform at the Science for Life Laboratory. DDD: Drug discovery and development.

The objective of most academic drug discovery centers (a collection of which can be found at the Academic Drug Discovery Consortium; [36]) [37] is to help identify novel targets and develop accompanying deep biological expertise within the academic community and to help de-risk these target by developing a lead-like small molecule or human antibody. Many universities in the USA [38] and in Europe [39] have created such centers in order to meet a new reality, where more and more of the early pharmaceutical research is ‘outsourced’ from big pharma to academia and biotech companies. Although we have only been operational for 3 years, a short time frame for pharmaceutical development, we see a large interest from global pharmaceutical companies to get a glimpse on the science behind our projects (Figure 1). However, in the Swedish system an academic researcher owns the rights to commercialize his or her own inventions without the need to engage the university; this ‘professor's privilege law’ is quite unique world-wide and the consequences were outlined in a previous publication [6]. The professor's privilege means that the academic principal investigator (PI) is the owner of the IP generated by the research performed at the university and decides on the commercialization path for his/her project; some prefer to seek venture capital funding to start their own company, while others instead prefer to partner the project with an established pharma partners. We have encountered both during the work with our projects. In one case, the principal investigator founded a company (Glionova therapeutics) whose lead molecule was backed for GLP toxicology studies by a consortium of investors including Novo Seeds, Healthcap, Industrifonden and others. In a more recent example, the PI decided to establish a research agreement with the Polish pharma company Selvita, already after which the first patent application was submitted. As a third option, which has happened in two cases so far, the academic PI has decided to progress their projects to the clinic within the academic setting; in one example this concerned a re-purposing, but in another case the PI has funded GMP manufacturing, GLP toxicology, investigational new drug (IND) filing and approval through research grants.

## Future perspective

SciLifeLab DDD is important for the identification of future therapeutic targets and drugs stemming from the Swedish academic society. A closer interaction between scientific research in academy and applied research in biopharma/pharma is needed for the optimal progression of findings done in basic science to clinical applications. It is therefore expected that an organization like the SciLifeLab DDD will facilitate the exposure of new drug opportunities for further investments in the drug development phase to the clinic. SciLifeLab DDD is a key component as a drug discovery and development tool-box for Swedish life science and for fruitful collaborations between Swedish academic, biopharma/pharma projects. In the future, it is anticipated that the SciLifeLab DDD will become even more an international organization interacting/collaborating with both international academic and biopharma/pharma organizations.

**Executive summary**

- The Science for Life Laboratory Drug Discovery and Development (SciLifeLab DDD) was established by a group of scientists with experience from both academy and biopharma/pharma in 2014. The SciLifeLab DDD has subsequently been developed to a high international industry standard infrastructure which support academic projects to reach preclinical proof-of-concept.
- The SciLifeLab DDD with its management and top science experts of its ten facilities have developed a broad Swedish and international contact network.
- The high scientific standard of the ten SciLifeLab DDD expert facilities is demonstrated by publications and intellectual properties provided to academic scientists. It should be noted that this level was achieved within three years.
- The improvement of the processes of small molecules and antibodies to reach the level of preclinical proof-of-concept increases the importance of academic research as a source for novel drug projects as well as of biopharma/pharma discovery research and development. Such improvements are most appreciated for the further improvement of medical research and development activities.
- The project portfolio and the current state of the drug and development projects of Q1 2017 is shown in Figure 1.
